# Equine influenza vaccination in the UK: Current practices may leave horses with suboptimal immunity

**DOI:** 10.1111/evj.13377

**Published:** 2020-12-09

**Authors:** Amie Wilson, Gina Pinchbeck, Rachel Dean, Catherine McGowan

**Affiliations:** ^1^ Department of Equine Clinical Science Institute of Veterinary and Ecological Sciences University of Liverpool Neston Cheshire UK; ^2^ Vet Partners Leeman House York UK

**Keywords:** horse, adverse, hesitancy, influenza, outbreak, UK, vaccination

## Abstract

**Background:**

Vaccination is integral to preventive healthcare. Despite numerous guidelines on equine vaccination, evidence of current vaccination practices is lacking.

**Objectives:**

To describe current vaccination practices advised by vets treating horses in the United Kingdom (UK) and compare practices with manufacturer datasheets and current guidelines.

**Study design:**

Cross‐sectional survey.

**Methods:**

An online questionnaire was distributed using email addresses acquired through professional registration listings and social media, targeting vets who treat horses in the UK. The questionnaire collected demographic data and information regarding vaccination practices and vaccine hesitancy. Descriptive statistical analysis was performed.

**Results:**

Questionnaires were completed by 304 UK vets working with horses used for leisure (97.4%, n = 296/304), competition (86.2%, n = 262/304), stud‐work (47.7%, n = 145/304) and racing (40.5%, n = 123/304). Variation was identified in vaccine protocols for competition and noncompetition horses. Fifty‐seven per cent (n = 170/298) of respondents reported variation in advised ‘booster’ frequency; most commonly (n = 118) advising a 6‐monthly vaccination in competition horses and annual vaccination in noncompetition horses. Most common vaccination guidelines volunteered were British Horseracing Authority (68.8%, n = 172/250) and Federation Equestre Internationale (66.4%, n = 166/250). Most vaccination practices were not consistent with datasheet guidance. Only 7.7% (n = 23/300) of respondents complied with datasheet timeframes between the second and third vaccination. Adverse events following vaccination in the previous year were encountered by 66% (n = 199/304) of respondents, representing 2760 adverse events; but only 526 (19.1%) cases were reported to the Veterinary Medicines Directorate. Most common reactions were transient, including stiffness (931), localised swelling (835), lethargy (559) and pyrexia (355). 86.4% respondents reported vaccine hesitancy from horse owners, most commonly due to perception of over‐vaccination, cost and concern regarding adverse events.

**Main limitations:**

Potential selection, respondent and recall bias. The recent Equine Influenza (EI) and Equine Herpes Virus (EHV) outbreaks in the UK may have altered responses.

**Conclusions:**

Current equine vaccination practices, although complying with competition rules, are mostly noncompliant with datasheet guidelines, potentially risking suboptimal immunity.

## 1 INTRODUCTION

Vaccination is an integral component of preventive medicine in equine practice. Equine vaccination is most commonly performed against equine influenza (EI) and tetanus, with an owner‐based survey reporting 71.3% horses were vaccinated for both EI and tetanus in Great Britain^1^ with lower vaccination rates in ponies, retired and companion animals. This study, however, is likely to be an overestimation due to respondent bias, and although sufficient data in this area are lacking, crude estimates which take into account vaccine manufacturer sales and the estimated horse population in the United Kingdom (UK) give an approximate figure of only 30% horses that are vaccinated (R. Newton, personal communication, 2020).

There are multiple sources of guidance and regulations on equine vaccination in the UK, particularly for EI; including various competition, riding club and racing rules,^2^, ^3^, ^4^ in addition to organisations including the World Health Organisation for Animal Health (OIE).^5^ These guidelines often differ from each other and also from the manufacturer's published datasheets,^6^ as summarised in Table 1.

**Table 1:** Comparison of (A) datasheet antigen listings and protocols for equine influenza (EI) vaccination intervals (for Equip F, Equilis Prequenza and ProteqFlu). (B) equestrian body guidelines for antigen inclusion and EI vaccination intervals (British Equestrian Foundation (BEF), Federation Equestre Internationale (FEI) and British Horseracing Authority (BHA) and World organisation for Animal Health (OIE) and (C) respondents vaccination practices

Recommendations	(A)			(B)				(C)
	Equip F (Zoetis)	Equilis Prequenza (MSD Animal Health)	ProteqFlu (Boehringer Ingelheim)	BEF BE/BD/BS/PC/BHS/RDA/HPA etc^4^	FEI^3^	BHA^2^	OIE World Health Organisation for Animal Health—OIE expert surveillance panel April 2020 update^5^	Results from this survey: percentage of respondents with vaccine schedules compliant with the data sheet
Antigens included	Influenza/A/eq/Newmarket/77 (H7N7) Influenza/A/eq/Boulange/91 (H3N8) Influenza/A/eq/Kentucky/98 (H3N8)	Influenza/A/eq‐2/SouthAfrica/4/03 Influenza/A/eq‐2/Newmarket/2/93	Influenza/A/eq/ Ohio/03 (H3N8) Influenza/A/eq/Richmond/1/07 (H3N8)	/	Clade 1 & Clade 2 sublineage	/	It is not necessary to include an H7N7 virus or an H3N8 virus of the Eurasian lineage in vaccines. Vaccines should contain both clade 1 and clade 2 viruses of the Florida sublineage. Clade 1: A/eq/South Africa/04/2003‐like or A/eq/Ohio/2003‐like viruses. Clade 2 continues to be represented by A/eq/Richmond/1/2007‐like viruses.	/
Age of 1st vaccination	> 5 months	>6 months *Can be administered at >4 months in the event of reduced colostrum intake, however, the primary course must be repeated at 6 months	5‐6 months *Can be administered at >4 months in the event of reduced colostrum intake, however, the primary course must be repeated at 5‐6 months	/	/	/	/	Equilis prequenza: 77.1% (n = 118/153) competing horses and 77.8% (n = 119/153) in noncompeting horses. ProteqFlu: 93.3% (n = 113/121) in all horses. Equip F: 100% (n = 7/7) compliance in all horses.
Interval of primary course. (1st‐2nd vaccine)	6 weeks	4 weeks	4‐6 weeks	21‐92 days (3 weeks to 3 months)	21‐92 days (3 weeks to 3 months)	21‐92 days (3 weeks to 3 months)	/	Equilis Prequenza: 21.6% (n = 33/153) in all horses. ProteqFlu: 48.8% (n = 61/125) in competing horses and 50.4% (n = 63/125) in noncompeting horses. EquipF: 14.3% (n = 1/7) for all horses
Interval for third vaccination. (2nd‐3rd vaccine)	5 months	5 months	5 months	150‐215 days (~5‐7 months)	<7 months	150‐215 days (~5‐7 months)	/	Equilis Prequenza: 5.2% (n = 8/153) for all horses. ProteqFlu: 8.8% (n = 11/125) for all horses. EquipF: 0% (n = 7/7) for all horses.
Interval for booster vaccination. (3rd‐4th onwards)	12‐15 months	<12 months	<12 months	Varies with organisation 12 months for BS/RDA BE/BD/BRC 12 months but within 6 months 21 days of competition HPA‐ at all times boosters must be given within 6 months 21 days COVID19 response‐ 12 months allowance‐ April 2020 onward	12 months but within 6 months 21 days on day of competition	6 months Feb‐May 2019 9 months May 2019‐April 2020 12 months COVID 19‐April 2020 onward	/	Equilis Prequenza: 100% (n = 153/153) in competing horses, and 99.3% (n = 152/153) in noncompeting horses. Proteqflu: 100% (n = 125/125) in all horses. EquipF: 100% (n = 7/7) in all horses.
Onset of immunity following primary course; OR/accepted interval post‐vaccination prior to competing	14 days	14 days	14 days	Varies with organisation BE/BD/HPA‐ 7 days PC/BRC–6 days BS—none stated	7 days	6 days	/	Equilis Prequenza: 9.8% (n = 15/153) ProteqFlu: 6.4% (n = 8/125) EquipF: 14.3% (n = 1/7)

Abbreviations: BE, British Eventing; BD, British Dressage; BS, British Showjumping; PC, Pony Club; BHS, British Horse Society; RDA, Riding for the Disabled Association; HPA, Hurlingham Polo Association.

With outbreaks of EI, and equine herpes virus (EHV) in the UK in 2019, the effectiveness of vaccination continues to be under close scrutiny. Equiflunet,^7^ a free online disease surveillance tool created by the Animal Health Trust, reported 228 laboratory confirmed outbreaks of EI in 2019, most of which affected multiple horses. Of concern were numerous reports of EI in vaccinated animals in the UK and internationally.^8^, ^9^, ^10^, ^11^ One factor in vaccination failure is noncompliance with the published vaccination guidelines available at the time which has been demonstrated in cats.^12^ However, to date, there has been a lack of data on current equine vaccination practices; whether veterinary advice is consistent with datasheet recommendations and which factors or available guidelines influence vets’ decision making.

Vaccine hesitancy is defined by a reluctance or refusal to be vaccinated or to have an individual vaccinated. It has been recently identified as one of the top 10 global threats by the World Health Organisation in 2019.^13^ This has not been described in the equine veterinary sector in the UK.

This survey aimed to describe current vaccination practices advised by vets treating horses in the UK and compare practices with current guidelines including the manufacturer's datasheet and industry guidelines. Further aims included gathering information regarding influential factors, including the impact of the recent EI outbreak, on vets’ vaccination practices, and the prevalence of adverse drug reactions following vaccination and vaccine hesitancy in horse owners.

## 2 MATERIALS AND METHODS

An online questionnaire was created using JISC software (JISC Online Surveys^©^, 2020). The questionnaire was first piloted to 6 veterinary surgeons to assess that the questions were answerable, and the software was effective. No changes were required following the pilot. The questionnaire link was distributed using practice email addresses acquired through the Royal College of Veterinary Surgeons (RCVS) ‘Find a Vet’ website (filtered for practices treating horses) and published on appropriate social media pages (Veterinary Voices and Veterinary Voices Equine). The survey was launched on 11 November 2019 and closed on the 12 February 2020; and was incentivised with entry into a prize draw for a gift voucher. The prize draw entry was linked to another questionnaire, enabling the original questionnaire to remain anonymised.

Informed consent prior to completion required participants to indicate that they agreed with the given information and that they agreed to take part in the study. Secondly, the survey had screening questions to ensure all participants were veterinary surgeons working in the UK who treated horses. The questionnaire was comprised of 8 sections; consent, screening, demographic information, vaccine choice, vaccine policy, adverse drug reactions relating to vaccination, experiences of vaccine hesitancy and case‐based examples. The questions were a variety of multiple choice, free text and grid style. (The questionnaire is available as Data S1).

Descriptive analyses were performed using JISC survey software, Microsoft XL 16.37 (2020) and IBM SPSS 25 (2017) software. Categorical variables were reported as absolute numbers and percentages with 95% confidence intervals. A comparison was made to the vaccine product used for each individual response to determine compliance with datasheet recommendations for that vaccine product. The open‐ended questions, where free text was given in response, were converted into categorical variables where appropriate. Not every respondent answered every question, therefore, the number of responses reported for each question varies and is stated throughout.

## 3 RESULTS

### 3.1 Respondents

The survey resulted in 304 valid responses from veterinary surgeons treating horses. Seventy‐four per cent respondents worked only with horses; others were mixed practitioners. 55.2% were less than or equal to 10 years graduated. The respondents’ workload consisted of a combination of leisure horses (97.4%), competition horses (86.2%), stud (47.7%) and racing (40.5%).

#### 3.1.1 Target diseases, protocols and products

**Figure 1:**
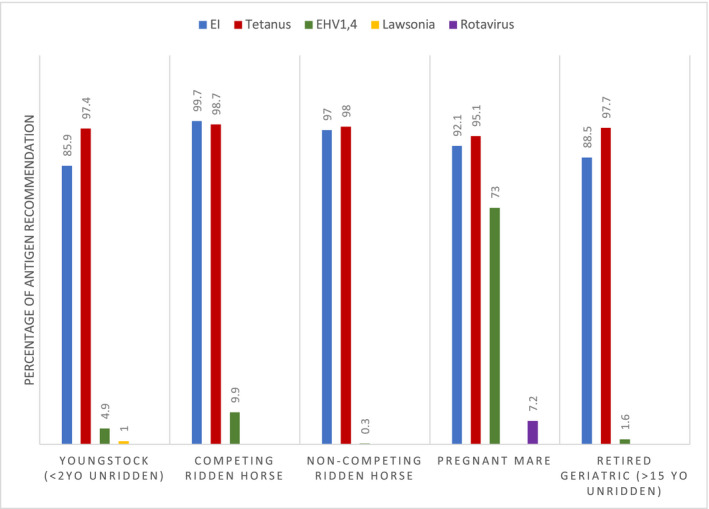
Frequency of antigen recommendation by 304 veterinary surgeons divided by equestrian groupings (by % of veterinary surgeons recommending)

In this survey, the conditions most commonly vaccinated against were EI, tetanus and Equine Herpes (types 1 & 4). Tetanus was the most commonly advised vaccine in noncompeting ridden horses (n = 298/304, 98.0%; 95% CI 95.8%‐99.1%), retired geriatric horses (n = 297/304, 97.7%; 95% CI 95.3%‐98.9%), youngstock (n = 296/304, 97.4%; 95% CI 94.9%‐98.7%) and pregnant mares (n = 289/304, 95.1%; 95% CI 92.0%‐97.0%). EI and tetanus were most common diseases vaccinated against in competing ridden horses (EI; n = 303/304, 99.7%; 95% CI 88.2%‐99.9%. Tetanus; n = 300/304, 98.7%; 95% CI 96.7%‐99.5%). Vaccines protecting against the pathogens EHV1&4 were commonly used in pregnant mares (n = 222/304, 73.0%; 95% CI 67.8%‐77.7%). Vaccines protecting against Rotavirus were advised for use in pregnant mares by 7.2% (n = 22/304; 95% CI 4.8%‐10.7%) practitioners (Figure 1).

**Figure 2:**
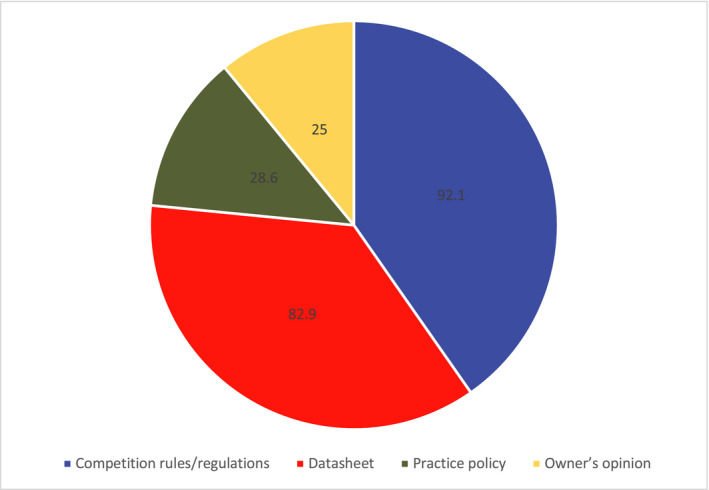
Factors influencing vaccination protocols chosen by UK veterinary surgeon respondents

When asked to indicate which factors influenced their equine vaccination protocol (Figure 2), most respondents were influenced by competition rules or regulations (92.1%, n = 280/304; 95% CI 88.5%‐94.6%). 82.9% indicated they were influenced by the licensed manufacturers’ datasheet (n = 252/304; 95% CI 78.3%‐86.7%). Other influential factors included: practice policy (n = 87/304, 28.6%; 95% CI 23.8%‐33.9%) and owners’ opinion (n = 76/304, 25%; 95% CI 20.5%‐30.2%).

**Figure 3:**
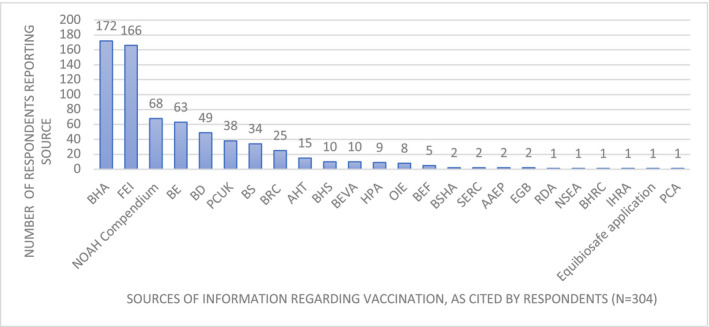
Respondent indicated guidelines for equine vaccination in horses, as cited by UK veterinary surgeon respondents. BHA, British Horseracing Authority; FEI, Federation Equestre Internationale; NOAH, National Office of Animal Health; BE, British Eventing; BD, British Dressage; PCUK, Pony Club United Kingdom; BS, British Showjumping; BRC, British Riding Club; AHT, Animal Health Trust; BHS, British Horse Society; BEVA, British Equine Veterinary Association; HPA, Hurlingham Polo Association; OIE, Office Internationale des Epizooties (World Organisation for Animal Health); BEF, British Equestrian Federation; BSHA, British Show Horse Association; SERC, Scottish Endurance Riding Club; AAEP, American Association of Equine Practitioners; EGB, Endurance Great Britain; RDA, Riding for the Disabled Association; NSEA, National Schools Equestrian Association; BHRC, British Harness Racing Club; IHRA, Irish Harness Racing Association; PCA, Pony Club Australia

When asked if they were aware of any vaccination guidelines, 89.4% (n = 269/301; 95% CI 85.4%‐92.4%) vets indicated awareness of 25 different sources of guidelines. These included a variety of competition, racing and riding club regulations as well as guidance from veterinary and public health groups such as OIE, AHT, AAEP (American Association of Equine Practitioners) and manufacturers’ datasheets (Figure 3).

**Figure 4:**
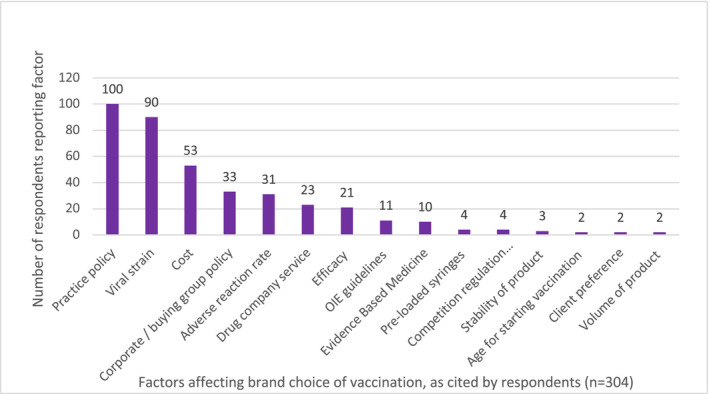
Factors affecting equine vaccine brand choice as cited by respondents (n = 304)

The most commonly used equine influenza vaccine products used were Equilis Prequenza (MSD Animal Health) (50.5%, n = 153/303; 95% CI 44.9%‐56.1%), ProteqFlu (Boehringer Ingelheim) (41.3%, n = 125/303; 95% CI 35.9%‐46.9%) and Equip F (Zoetis) (2.6%, n = 8/303; 95% CI 1.3%‐5.9%), with the rest of respondents using a combination of different products. When given the opportunity to comment in free text, this study's respondents stated that the main factors affecting vaccine brand choice included; practice (or corporate or buying group), decision (43.8%, n = 133/304; 95% CI 38.3%‐49.4%), coverage/viral strain (29.6%, n = 90/304; 95% CI 24.8%‐35.0%), cost (17.4%, n = 53/304; 95% CI 13.6%‐22.1%) and apparent rate of adverse reactions (10.2%, n = 31/304; 95% CI 7.3%‐14.1%). When referring to the effectiveness of the product, numerous free‐text responses referred to the most ‘up‐to‐date’ viral strain and 21 references were made specifically regarding clade of the vaccine, however, only 3.6% responses (n = 11/304; 95% CI 2.0%‐6.4%) referred to the OIE guidelines as an influential factor (Figure 4).

#### 3.1.2 Equine Influenza vaccine practices

Respondents’ recommendations for the age of first vaccination ranged from 6 weeks to 4 years old. The most commonly recommended age for starting vaccination was from 6 months old. 97.4% (n = 296/304; 95% CI 94.9%‐98.7%) respondents advised the same age of first vaccination for both competing and noncompeting animals. For the first vaccination age, 89.1% (n = 271/304; 95% CI 85.1%‐92.2%) of responses were compliant with datasheet recommendations in competition horses, and this was similar in noncompetition horses (89.5%, n = 272/304; 95% CI 85.5%‐92.4%). The most common reason for noncompliance was vaccinating earlier than 6 months with the Equilis Prequenza (MSD Animal Health) product*. Across all products used, 29.6% (n = 90/304; 95% CI 24.8%‐35.0%) respondents’ recommended protocols where first vaccination could be initiated in animals under 6 months of age.

(*Equilis Prequenza, MSD Animal Health is licensed for use from 4 months in foals with an insufficient colostrum intake, however, the datasheet recommends restarting a primary course at 6 months in this instance.)

Respondents’ recommendation for first to second vaccine interval ranged from 21 days to a maximum of 95 days. The most common interval stated was 21‐92 days for both competing and noncompeting horses, recommended by 42.7% (n = 128/300; 95% CI 37.2%‐48.3%) of respondents for competition horses and 41.7% (n = 125/300; 95% CI 36.2%‐47.3%) for noncompetition horses. This recommendation was compliant with many competition guidelines, but the range was greater than any of the datasheet recommendations of licensed vaccinations in the UK (typically 4‐6 weeks). Approximately a third of vets recommended 1st‐2nd vaccination intervals that were compliant with datasheet recommendations: 34% (n = 102/300; 95% CI 28.9%‐39.5%) and 35% (n = 105/300; 95% CI 29.8%‐40.6%) for competing horses and noncompeting horses respectively. Noncompeting horses and competing horse vaccination protocols were identical in 95% (n = 285/300; 95% CI 91.9%‐96.9%).

Recommended intervals between the second to third vaccines ranged from 90 to 250 days. The most common interval stated was 150‐215 days; recommended by respondents in 62.7% (n = 188/300; 95% CI 57.1%‐67.9%) competing horses and 60% (n = 180/300; 95% CI 54.4%‐65.4%) in noncompeting horses. Again, this recommendation was compliant with many competition regulations, but the range was greater than the datasheet recommendations of all licensed products (vaccination within 5 months or 150 days based on duration of immunity after the primary course). Only 7.7% (n = 23/300; 95% CI 5.2%‐11.2%) of responses for competition horse vaccination were potentially compliant with datasheets, as the majority of the respondents’ recommendations exceeded 5 months. This was largely similar in both competing and noncompeting horses with only 4.7% (n = 14/300; 95% CI 2.8%‐7.7%) variation between competition protocols and noncompeting protocols.

‘Booster’ vaccination or repeat vaccination following primary vaccination course represented the greatest variation between competition and noncompetition horses. Fifty‐seven per cent (n = 170/298; 95% CI 51.4%‐62.5%) of respondents advised differing protocols for noncompeting horses. The most common interval was 6 monthly (60.7%, n = 181/298; 95% CI 55.1%‐66.1%) for competition horses and annual (70.1%, n = 209/298; 95% CI 64.7%‐75.0%) for noncompeting horses.

**Figure 5:**
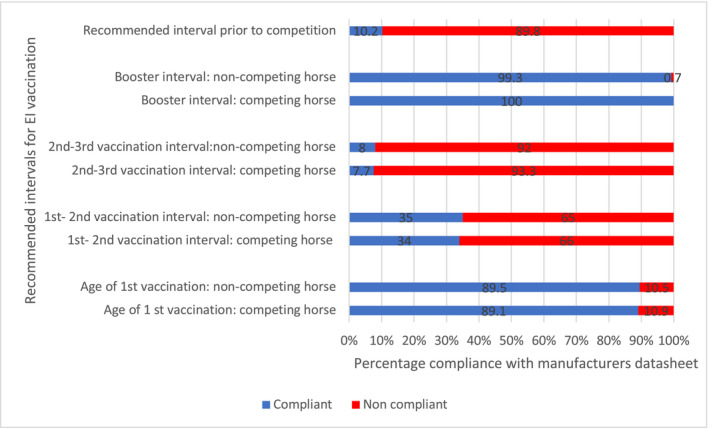
Comparison of respondent‐indicated equine influenza vaccination protocols with manufacturer's datasheet

Respondents were asked to indicate the interval that they advise following the primary course of vaccinations and prior to attending an event. Only 10.2% (n = 26/254; 95% CI 7.1%‐14.6%) of respondents complied with the datasheet recommendation of a minimum of 14 days prior to attendance, all other respondents advised return to competition prior to this (Figure 5).

#### 3.1.3 Adverse drug reactions following vaccination

Over half of respondents (65.5%, n = 199/304) encountered at least one adverse drug reaction (ADR) following vaccination in the previous 12 months. Respondents then provided an estimation of the number of ADRs encountered for each type of clinical presentation in the previous 12 months (Table 2). Therefore, based on an estimation from this survey, a total of 2,760 ADRs were encountered in the last 12 months by 199/304 respondents. However, 107/199 (53.8%, 95% CI 46.8%‐60.6%) respondents estimated that they had actually reported an ADR in only 526 of the 2760 cases (19.1%, 95% CI 17.6%‐20.6%). This indicated significant under reporting of encountered ADRs. The study did not record to whom these events were reported to.

**Table 2:** Adverse drug reactions (ADR) encountered by veterinary surgeon respondents in the UK in the last 12 months. (n = 304)

Adverse drug reaction following vaccination	Total estimated number of cases	Number of respondents (%) reporting stated ADR
Muscle stiffness at site of administration	928	162 (81.4%)
Transient swelling at site of administration	837	175 (87.9%)
Transient lethargy	559	123 (61.8%)
Transient pyrexia	355	102 (51.3%)
Abscessation	43	31 (15.6%)
Lack of efficacy of vaccination	36	6 (3.0%)
Urticaria	1	1 (0.5%)
Profound poor performance	1	1 (0.5%)

#### 3.1.4 Vaccine hesitancy

The majority (86.4%, n = 261/302; 95% CI 82.1%‐89.8%) of respondents had encountered some aspect of vaccine hesitancy or reluctance to vaccinate from owners. When asked to describe the frequency of encountering vaccine hesitancy, 298/304 of the survey participants responded, of whom 57.5% (n = 150/298; 95% CI 44.7%‐56.0%) encountered it rarely (less than annual), 45.6% (n = 119/298; 95% CI 34.5%‐45.6%) encountered it sometimes (less than monthly) and 11.1% (n = 29/298; 95% CI 6.9%‐13.6%) encountered it often (more than monthly). The most common reasons for vaccine hesitancy in horse owners encountered by respondents were as follows unnecessary need of vaccination, previous or anticipated ADR, side effects and lack of effectiveness (Table 3).

**Table 3:** Reasons for vaccine hesitancy in horse owners as described by veterinary respondents (n = 292)

Reason for vaccine hesitancy	Percentage reported (95% confidence intervals)	Number reported (n = x/292)
Unnecessary need	77 (71.5‐81.2)	224
Cost	67.4 (61.5‐72.3)	196
Previous adverse reaction	56.7 (50.8‐62.1)	165
Anticipated adverse reaction	47.1 (41.3‐52.6)	137
Side effects	39.2 (33.6‐44.7)	114
Lack of efficacy	12 (8.7‐16.2)	35
Other^a^	4.8 (2.9‐7.9)	14
Reason unstated	4.5 (2.6‐7.5)	13

^a^ Including: Needle shy horses, owners of older horses on private property, Shetland/miniature pony owners and aversion to 6 monthly vaccination [with perceived over‐vaccination].

#### 3.1.5 Case‐based scenarios

When attending a horse with a cough and pyrexia, 37.3% (n = 113/303, 95% CI 32.0‐42.9) of respondents would always perform a nasopharyngeal (NP) swab for EI diagnostic surveillance, whereas 52.8% (n = 160/303, 95% CI 47.2%‐58.4%) would perform a swab sometimes and 8.3% (n = 25/303, 95% CI 5.7%‐11.9%) would only perform an NP swab if the horse had not been vaccinated for EI. 2.3% (n = 7/303, 95% CI 1.1%‐4.7%) of respondents declared they would never perform an NP swab for EI surveillance in this scenario.

In the event of a noncompeting horse lapsing the annual influenza vaccination by less than 30 days; 18.8% (n = 57/304, 95% CI 14.8%‐23.5%) of respondents would advise restarting the primary course (A), 14.8% (n = 45/304, 95% CI 11.3%‐19.2) would advise administering a single booster vaccination (B) and 63.5% (n = 193/304, 95% CI 57.9‐68.7) would perform A or B dependent on the client's decision. However, if the annual influenza vaccination had lapsed by more than 30 days; 58.2% (n = 177/304, 95% CI 52.6%‐63.6%) vets would advise restarting the primary course (A), 1.3% (n = 4/304, 95% CI 0.5%‐3.3%) would advise administering a single booster vaccination (B) and 35.5% (n = 108/304, 95% CI 30.4%‐41.1%) would perform A or B dependent on the client's decision.

#### 3.1.6 Change in practice following 2019 EI outbreak

Of the respondents, 63.2% (n = 192/304, 95% CI 57.6%‐68.4%) had permanently changed their practice following the 2019 EI outbreak, 18.8% (n = 57/304, 95% CI 14.8%‐23.5%) had changed their practice temporarily and resumed previous practice and 18.1% (n = 55/304, 95% CI 14.2%‐22.8%) had not changed their practice. Of those who changed their practice; 97.2% (n = 242/249, 95% CI 94.3%‐98.6%) changed frequency of administration, with 57.4% (n = 139/242, 95% CI 51.1%‐63.5%) of those changing to 6 monthly ‘booster’ vaccination, 10.4% (n = 26/249, 95% CI 7.2%‐14.9%) changed brand of vaccination and 3.6% (n = 9/249, 95% CI 1.9%‐6.7%) used different antigens; most commonly by incorporating EI vaccination into protocols for geriatric/unridden animals (as opposed to tetanus alone previously).

## 4 DISCUSSION

This study provides insights into equine vaccination practices performed by vets in the UK. As expected,^14^ vaccination against EI is almost universally (≥95%) recommended by vets working across different areas of equine practice and across horses representing competing, noncompeting youngstock and pregnant mares as well as retired horses. However, despite EI vaccination being recognised as important, most reported vaccination practices were not consistent with datasheet guidance, potentially leaving horses with suboptimal immunity.

One reason for poor compliance with manufacturers’ datasheets and OIE recommendations may be the number and variability of vaccination guidelines, recommendations and regulations available to horse owners and vets. This study identified 25 different types of resource in use by 304 veterinary surgeons and demonstrated a greater consistency with competition guidelines than the datasheet advice. It is not surprising that there is variation in vaccine practices among veterinary surgeons when there is such disparity among different vaccine regulations.

This study demonstrated that 29.6% of respondents may perform first vaccination (V1) before 6 months of age, although age of first vaccination has been correlated with the presence of maternally derived antibodies and both of these factors may impair the establishment of an effective humoral response.^15^ Foals receiving V1 at 6 months of age had significantly higher antibody levels (optimal immunity) 1 month after V3 than foals receiving V1 at 4 months (suboptimal immunity). Differing levels of immunity can result in alteration in viral shedding and clinical signs. The single radial haemolysis (SRH) assay of antibodies is predictive for disease severity. SRH antibody levels greater than 85 mm^2^ are associated with reduced clinical signs and levels between 120 and 154 mm^2^ are associated with resistance to clinical disease.^16^

All manufacturers of authorised EI products within the UK advise a 3rd vaccination (V3) 5 months following the 2nd vaccination (V2) due to the duration of immunity (5 months) following V2.^6^ However, this is not in accordance with competition guidelines which largely state V3 can be administered from 5 to 7 months. FEI^3^ offers differing advice to BHA,^2^ and BEF^4^ as V3 must only be administered within 7 months of V2. Although unlikely, V3 can in theory be administered 1 day following V2 in accordance with the FEI rules, and up to 7 months after V2 with FEI/BHA/BEF. This is not compliant with the datasheets advice and Cullinane et al^17^ demonstrated that prolonged intervals between V1‐V2 and V2‐V3 (as allowed by the BHA/FEI/BEF) results in immunity gaps which prolong periods of susceptibility to EI in vaccinated horses. Investigation of the 2003 EI outbreak in Newmarket found an increased risk in horses that were not vaccinated in the last 3 months,^8^ demonstrating the importance of duration of immunity following vaccination. This was supported by findings from investigation of other outbreaks in Ireland and the UK, which found an increased risk in horses not vaccinated within the last 6 months.^10^, ^18^ However, there was limited indication of an immunity gap when a whole inactivated ISCOMatrix adjuvanted EI and tetanus vaccine (Equilis Prequenza, MSD Animal Health Te) were used in 7 Welsh ponies which were experimentally infected, 152 days after V2.^19^ This demonstrates that different vaccine technology along with appropriate vaccine schedules may help to address the immunity gap.

All equine influenza vaccine manufacturers within the UK state 14 days as the interval following primary course to onset of immunity, in line with research demonstrating peak of antibody level was reached 2‐4 weeks post vaccination,^20^ however, many competition guidelines allow a shorter interval following vaccination to attendance at an event. Only 10.2% (n = 26/254) respondents were consistent with the datasheet recommendation of a minimum of 14 days prior to attendance. This potentially enables horses to attend events prior to onset of immunity. This is likely to be more important in younger horses that have only received a primary course, or those without a history of regular vaccinations.

In other efforts to promote EI immunity following vaccination, concurrent vaccination of horses with EI and EVH1,4 results in an improved response to EI vaccination.^21^ This may be worth consideration in a ‘high risk’ competition animal as this study demonstrates that currently EHV1,4 vaccination is only performed in 9.9% competing horses.

Many veterinary surgeons performing equine vaccination indicated limitations in individual choice over the brand of vaccination used. The brand of vaccine impacts the effectiveness of vaccination, as different EI vaccinations contain differing antigens. This may affect the ability of practitioners to respond to the OIE recommendations.^5^ The OIE Expert Surveillance panel meet annually in order to provide guidance on currently circulating EI strains to inform vaccine usage, with the conclusions and recommendations then made available online. The current OIE guidelines^5^ are met by one authorised product in the UK (ProteqFlu, Boehringer Ingelheim), acknowledged (although not enforced) by the FEI, used by 41.3% of respondents. Following the Australian outbreak in 2007,^22^ a judicial enquiry highlighted the importance of utilising the OIE guidelines to inform the most appropriate antigen strains for vaccination of imported horses. The importance of adhering to OIE guidelines was reiterated following review of the 2018 Argentinian EI outbreak in vaccinated horses,^11^ where a vaccine breakdown was suspected due to ‘out‐of‐date EIV strains’ in vaccines. However, there was a recent outbreak of Florida Clade 1 in France despite compliance with OIE vaccination guidelines,^23^, ^24^ therefore, a multifactorial approach to EI vaccination is required. Further antigenic analysis in 2020 from the OIE is awaited.

Another concern highlighted by this study is the low level of ADR reported despite frequent encounters of adverse events. Participants reported encountering 2760 adverse events in the last 12 months, of those events only 19.1% were formally reported. This is an area which requires improvement as a profession in order to provide necessary feedback to pharmaceutical companies and the veterinary medicines directorate (VMD) for drug safety. The concern about the risk of ADR appears to be a significant contributing factor when encountering vaccine hesitancy in horse owners. The risk of ADR was the most common reason given for refusal of Hendra vaccination by Australian horse owners.^25^

Vaccine hesitancy (VH) has been defined by the WHO as “delay in acceptance or refusal of vaccines despite availability of vaccination services” and was categorised as one of the top 10 threats to global health in 2019.^13^ This study identified vaccine hesitancy or reluctance occurring in owners in the equine sector, with 11.1% practitioners encountering VH frequently. A recent meta‐synthesis study of childhood VH^26^ outlines similar themes of causation of VH to our equine study such as concern regarding side effects, and mistrust of health professionals, pharmaceutical companies and the information which they deliver. With regard to childhood vaccination, the doctor's advice has been shown to be the most important predictor of vaccine acceptance.^27^, ^28^ Awareness of this growing problem is, therefore, vital in the veterinary field, in order to address the underlying issue, as reduced uptake of vaccination will in turn affect our ability to promote herd immunity. Further research exploring horse owners’ perception of vaccination is required.

As this study was a cross‐sectional questionnaire requiring voluntary uptake, there was the potential for selection bias of the respondents. The ADR section and vaccine hesitancy section of the survey required respondents to estimate the frequency of hesitancy and adverse events encountered, therefore, may not be entirely accurate due to recall bias. A prospective study based on vaccination protocols would be welcome. It is possible that the recent EI and EHV1,4 outbreaks in the UK just prior to this survey could have affected the responses given. In an attempt to manage the effect of the recent EI outbreak on vaccination protocols, respondents were asked to report any changes in their practice in the last 12 months. In light of the EI outbreak, many competition guidelines altered their ruling to reduce the duration between booster vaccinations due to evidence of waning immunity over time since last vaccination.^8^, ^10^, ^18^, ^29^ These changes were supported by the change in practice of vets in this study, with 97.2% of respondents increasing the frequency of vaccination, most commonly (57.4%) to 6 monthly.

In addition to this, there was a human health pandemic (COVID19) at time of authorship, though following closure of the survey, which also has implications for equine vaccination. The RCVS and British Equine Veterinary Association (BEVA) advised against routine vaccination during the period of lockdown, and as a consequence many competition guidelines were relaxed to allow annual vaccination. This added a further level of complexity for practitioner's decision‐making regarding vaccination, and vets need to be aware of the duration of immunity stated by the manufacturers’ datasheet as horses vaccinated outside this schedule may not be fully protected.

In conclusion, this study has identified a lack of compliance with manufacturers’ datasheets that may promote immunity gaps leading to a reduction in the effectiveness of vaccination programs within the UK equine population. Such gaps may increase the risk of EI outbreaks, even among competition animals complying with the competition's ruling. Updating competition requirements to one strategy across all equestrian disciplines could reduce the number of differing guidelines and, in turn, improve equine welfare.

## CONFLICT OF INTERESTS

MSD (source of funding) produce a range of equine vaccinations; however, they were not involved in the development of the survey, and they did not have access to the results.

## AUTHOR CONTRIBUTIONS

A. Wilson contributed to study execution, data analysis and interpretation and writing of the manuscript. R. Dean assisted A. Wilson with application for the bursary, and piloting of the survey. G. Pinchbeck and C. McGowan assisted with survey development, data analysis and writing of the manuscript. All authors have approved the final manuscript.

## Ethical animal research

Ethics approval was obtained through the University of Liverpool Veterinary Ethics Committee (VREC838).

## Owner informed consent

Completion of the questionnaire was taken as participant consent.

## Data Accessibility statement

The data that support the findings of this study are available on request from the corresponding author. The data are not publicly available due to privacy or ethical restrictions.

### Peer Review

The peer review history for this article is available at https://publons.com/publon/10.1111/evj.13377.

## Supporting information

DataS1
