# Supplementary material for: Equine influenza vaccination in the UK: Current practices may leave horses with suboptimal immunity
Source: Equine Vet J. 2020 Dec 9;53(5):1004–14. doi: 10.1111/evj.13377 (PMC8451788; doi:10.1111/evj.13377)
Supplement: Supplementary file 1 — DataS1 [file EVJ-53-1004-s001.pdf]

Equine Veterinary Journal BEVA

Equine Vaccination Questionnaire

Participant Information Sheet

You are being invited to participate in a research study. Before you decide whether to participate, it is important for you to understand why the research is being done and what it will involve. Please take time to read the following information carefully. Researcher contact details are listed below if you have any further questions or if you would like more information.

We would like to stress that you do not have to accept this invitation and should only agree to take part if you want to.

Thank you for taking the time to read this.

What is the purpose of the study?

We are studying the equine vaccination protocols used by veterinary surgeons in the UK. Currently there are multiple sources of vaccination guidelines and rules for equine vaccinations, therefore we would like to gain a better understanding of what influences vets' decision making when vaccinating horses in practice.

We will use the data to report on the current equine vaccine practices in the UK.

Why have I been chosen to take part?

You are being invited to take part because you are a veterinary professional working with horses in the UK.

Do I have to take part?

Participation is voluntary and you do not have to take part in this study. You are free to withdraw at any time until you have selected the ‘finish’ button on the final page of the questionnaire. After this point, as the questionnaire is anonymous, we will not be able to identify your responses and therefore cannot remove them. You do not have to give a reason if you do not wish to take part.

What will happen if I take part?

If you decide to take part you will need to complete an **online questionnaire, with 20 questions which will take around 5-10 minutes**. Your answers will be anonymous and we will not contact you for any follow up questions, though free text within your answers could be quoted in future publications or presentations relating to this research.

How will my data be used?

Your email address if you choose to leave it will only be used for contact if you win the prize draw. More detail regarding processing of this data should you choose to leave it and GDPR can be found on the relevant page of the questionnaire.

Expenses and/or payments

No expenses, payments or reimbursements will be made for participation in this study. **You can choose to be entered into a prize draw for the chance to win a £50 John Lewis voucher**. To enter into the prize draw you will be required to leave your email address, this is completely voluntary. Your email address will not be used in the analysis of results. Winners will be contacted following completion of the study.

Are there any risks in taking part?

There are no direct risks associated with taking part in this questionnaire.

Are there any benefits in taking part?

There are no direct benefits associated with taking part in this questionnaire, but we will use the data to improve knowledge regarding equine vaccination practices in the UK.

What will happen to the results of the study?

The results will be used to improve awareness of current equine vaccination practices. We will also aim to present them at a relevant conference and publish them in an easily accessible journal (e.g Veterinary Record or Equine Veterinary Journal).

What will happen if I want to stop taking part?

If you want to stop taking part in this study you can withdraw at any time until you have selected ‘finish’ at the end of the questionnaire. After this your answers will be anonymous and therefore we will not be able to withdraw your information.

What if I am unhappy or if there is a problem?

If you are unhappy, or if there is a problem, please feel free to contact the researcher listed below and we will try to help. If you remain unhappy or have a complaint which you feel you cannot communicate directly to the researchers then you should contact the Research Governance Officer on 0151 794 8290 (ethics@liv.ac.uk). When contacting the Research Governance Officer, please provide details of the name or description of the study (so that it can be identified), the researchers involved, and the details of the complaint you wish to make.

Who can I contact for further details?

Amie Wilson

University of Liverpool

Leahurst Campus,

Chester High Road,

CH643TU

Email: A.Wilson3@liverpool.ac.uk

Have you read the above information and do you consent to participate in this study? ✱ *Required*

- ○ Yes, I have read the above information and I consent to participating in this study.

Screening

Are you a qualified veterinary surgeon working in the UK?

Section A : Introduction

How many years graduated are you?

- 0-5
- 6-10
- 11-15
- 16-20
- 21-25
- 26-30
- 30+

What type of veterinary practice do you perform? ✱ *Required*

- 100% Equine
- Mixed practice
- I do not treat horses

What proportion of your workload is equine?

Please enter a whole number (integer).

Please make sure the number is between 1 and 99.

What does your equine workload consist of? (Tick all that apply)

- Leisure/Pleasure horse
- Racing
- Sport/Competition horse
- Stud
- Hunting
- Other

If you selected Other, please specify:

Section B: Vaccine Choice

Which influenza vaccine do you currently use?

- Equip F (Zoetis)
- Equilis Prequenza (MSD)
- ProteqFlu (Boehringer Ingelheim)
- I use a combination of different products
- Other

If you selected Other, please specify:

What main factor affects your vaccine brand choice?

Section C: Vaccination Policy

Which of the following influence your vaccination protocol? (Tick all that apply.)

- ☐ Datasheet recommendations
- ☐ Competition rules/regulations
- ☐ Owner influence
- ☐ Practice policy
- ☐ Other

If you selected Other, please specify:

Are you aware of any equine vaccination guidelines?

- ○ Yes
- ○ No

If so, please list any guidelines you are aware of.

Which diseases do you routinely vaccinate against? (Tick all that apply)

	Vaccination *Optional*				
	Equine Influenza	Tetanus	Equine Herpes Virus (1&4)	Other	If you selected Other, please specify:

Youngstock (<2yo, not ridden)	☐	☐	☐	☐	
Competing ridden horse	☐	☐	☐	☐	
Non-competing ridden horse	☐	☐	☐	☐	
Pregnant mare	☐	☐	☐	☐	
Retired geriatric (>15yo)	☐	☐	☐	☐	

What is your **current** equine **influenza** vaccination protocol for competing and non competing horses?
Note- We will discuss any change in practice in section G

	Competition horse	Non-competing horse
Age of first vaccination		
Interval for 1st-2nd vaccination of initial course		
Interval for 2nd - 3rd vaccination of initial course		
Advised frequency of vaccination following initial course		

What interval do you advise following equine influenza vaccination and prior to attendance at a competition? (Please assume the vaccination schedule is appropriate prior to this point.)

Section D: Adverse reactions following vaccination

Have you had any adverse drug reactions (ADR's) **following vaccination** in the last 12 months?

- Yes
- No

Which of the following adverse reactions have you seen in the last 12 months following vaccine administration? (Tick all that apply and please give estimate of each type)

- Transient pyrexia
- Transient lethargy
- Transient swelling at site of administration
- Abscessation at site of administration
- Muscle stiffness at site of administration
- Lack of efficacy of vaccine
- Other

If you selected Other, please give further details regarding clinical signs and frequency of cases with this presentation:

How many cases have you seen with transient pyrexia in the last 12 months?

Please enter a whole number (integer).

How many cases have you seen with this transient lethargy in the last 12 months?

Please enter a whole number (integer).

How many cases have you seen with transient swelling at the site of injection in the last 12 months?

Please enter a whole number (integer).

How many cases have you seen with abscessation at site of administration in the last 12 months?

Please enter a whole number (integer).

How many cases have you seen with muscle stiffness at site of administration in the last 12 months?

Please enter a whole number (integer).

How many cases have you seen with lack of efficacy against the targetted disease in the last 12 months?

Please enter a whole number (integer).

Have you reported an Adverse Drug Reaction (ADR) **following vaccination** in the last 12 months?

- Yes
- No

How many ADR's **following vaccination** have you reported in the last 12 months?

Section E: Vaccine hesitancy

Vaccine hesitancy is a reluctance or refusal to be vaccinated or to have children/animals vaccinated.

It has been recently identified as one of the top ten global threats by the World Health Organisation in 2019. We are interested to see if this is an emerging issue in equine practice.

Have you ever experienced any issues with vaccine hesitancy (owners' reluctance or refusal to have their animal vaccinated) despite veterinary advice?

- ◯ Yes
- ◯ No

How often do you encounter this problem?

- ◯ Rarely (<Annually)
- ◯ Sometimes (<Monthly)
- ◯ Often (>Monthly)

When you encountered vaccine hesitancy, what were the most common client concerns? (Tick all that apply)

- ☐ Cost
- ☐ Lack of efficacy
- ☐ Unnecessary need
- ☐ Side effects
- ☐ Anticipated adverse reaction
- ☐ Previous experience of adverse reaction
- ☐ Not stated
- ☐ Other

If you selected Other, please specify:

Please give further details where possible.

Section F : Case examples

You are called to a pyrexic horse with a cough- would you perform nasopharyngeal swabbing for Influenza diagnostic surveillance on this horse?

- Always
- Only if the animal is unvaccinated
- Sometimes
- Never
- Other

If you selected Other, please specify:

An adult horse who does not compete but has had regular vaccinations with your practice has lapsed the annual Influenza vaccination by **less than** 30days, do you advise...

- A) Restart the primary vaccination course
- B) Give a single vaccination, acting as an annual booster
- A) or B) Based on owners opinion
- Other

If you selected Other, please specify:

An adult horse who does not compete but has had regular vaccinations with your practice has lapsed the annual Influenza vaccination by **more than** 30days, do you…

- A) Restart the primary vaccination course
- B) Give a single vaccination, acting as an annual booster
- A) or B) Based on owners opinion
- Other

If you selected Other, please specify:

Section G: Change in practice in last 12 months

In 2019, there was an outbreak of Equine Influenza in the racing industry, which resulted in media coverage and discussion regarding vaccination in the UK. This has led to tighter regulations by many competitions and equestrian businesses, we are interested to see how this may have influenced veterinary practice regarding vaccination.

Has your routine vaccination practice changed in the last 12 months?

- ○ Yes
- ○ Yes, but only temporarily during the outbreak, now returned to previous practice
- ○ No

How has your vaccination practice altered? (Tick all that apply)

- ☐ Changed frequency of administration
- ☐ Use of different antigens
- ☐ Change of vaccine brand
- ☐ Other

Please give further information about your change in practice

If you selected Other, please specify:

Final page

Thank you for completing the questionnaire, please click on the link below for your chance to win £50 John Lewis vouchers.

https://liverpool.onlinesurveys.ac.uk/prize-draw-for-equine-vaccination-survey

END OF QUESTIONNAIRE

Key for selection options

2 - Are you a qualified veterinary surgeon working in the UK?

Yes
No
